# Enzymatic Building‐Block Synthesis for Solid‐Phase Automated Glycan Assembly

**DOI:** 10.1002/anie.202008067

**Published:** 2020-10-02

**Authors:** Andrea Marchesi, Fabio Parmeggiani, João Louçano, Ashley P. Mattey, Kun Huang, Tanistha Gupta, Mario Salwiczek, Sabine L. Flitsch

**Affiliations:** ^1^ Manchester Institute of Biotechnology University of Manchester 131 Princess Street Manchester UK; ^2^ GlycoUniverse GmbH & Co KGaA Am Muehlenberg 11 14476 Potsdam Germany; ^3^ Department of Chemistry, Materials and Chemical Engineering, “G. Natta” Politecnico di Milano Via Mancinelli 7 20131 Milano Italy

**Keywords:** biocatalysis, chemoenzymatic synthesis, glycans, oligosaccharides, transgalactosylation

## Abstract

Automated chemical oligosaccharide synthesis is an attractive concept that has been successfully applied to a large number of target structures, but requires excess quantities of suitably protected and activated building blocks. Herein we demonstrate the use of biocatalysis to supply such reagents for automated synthesis. By using the promiscuous NmLgtB‐B β1‐4 galactosyltransferase from Neisseria meningitidis we demonstrate fast and robust access to the LacNAc motif, common to many cell‐surface glycans, starting from either lactose or sucrose as glycosyl donors. The enzymatic product was shown to be successfully incorporated as a complete unit into a tetrasaccharide target by automated assembly.

## Graphical abstract

In a chemoenzymatic approach to oligosaccharide synthesis, the scalability potential of enzymatic synthesis was exploited for the preparative synthesis of a relevant *N*‐acetyllactosamine disaccharide building block. This disaccharide was then applied successfully to the automated synthesis of a target oligosaccharide using the glycan synthesizer Glyconeer 2.1 (see scheme).

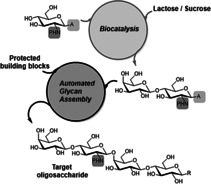

Oligo‐ and polysaccharides and their conjugates are important targets for chemical synthesis and a number of automated approaches have recently been developed in this field, including solid‐phase synthesis, HPLC‐assisted synthesis, fluorous‐tag solvent‐phase synthesis and artificial Golgi apparatus.[^1^, ^2^, ^3^, ^4^, ^5^, ^6^, ^7^, ^8^, ^9^] The formation of glycosidic linkages can either be achieved through biocatalysis using carbohydrate active enzymes or by chemical methods, which require selective protection and activation. The main strength of the enzymatic approach is scalability due to low numbers of steps, whereas chemical methods give access to a much broader range of natural and unnatural targets, while limited by the availability of complex, differentially protected building blocks. There have been some efforts to combine chemical and enzymatic methods, in particular incorporating chemically synthesized building blocks as starting materials for enzymatic strategies.[^10^, ^11^, ^12^]

Here we extend the chemoenzymatic toolbox, by demonstrating that biocatalysis can also be used to provide robust and short methods to access a building block for automated glycan assembly (AGA), thus combining the efficiency and scalability of biocatalysis where it is needed with the flexibility of chemical synthesis. We demonstrate this strategy for *N*‐acetyllactosamine (LacNAc), which is a widely recurrent motif in biologically relevant glycoconjugates, including human milk glycans, Lewis X antigens, N‐glycans, O‐glycans and bacterial polysaccharide capsules.[^13^, ^14^, ^15^, ^16^]

The target motif (Figure 1) was chosen as an example of a di‐LacNAc sequence that is isomeric to the common type 2 poly‐LacNac oligomer motif in glycoproteins and glycolipids. The target sequence has been reported in naturally derived oligosaccharide sequences[^17^, ^18^, ^19^, ^20^] but its structural and functional characteristics have not been explored as of yet. Compared to the more common motifs bearing a central GlcNAcβ1‐3Gal sequence the β1‐4 linkage is synthetically more challenging, because of the reduced reactivity of the axial hydroxyl group.^[21]^ In addition, whilst Type 1 (β1‐3) and 2 (β1‐4) polyLacNAc sequence can be generated enzymatically,[^22^, ^23^] the required enzyme with β1‐4 linkage selectivity is currently not available and hence this linkage has to be generated chemically. Due to its biological relevance, a variety of chemical synthetic strategies for the LacNAc motif **2** have been investigated,[^24^, ^25^, ^26^] often multistep with modest overall yield,[^24^, ^25^, ^26^] as exemplified by a nine‐step synthesis with an overall yield <30 %.^[26]^ Another approach to a multigram synthesis of LacNAc moiety is the convenient Heyns rearrangement starting from lactulose proposed by Wrodnigg et al.^[27]^ The enzymatic strategy we propose present some advantages in respect to the Heyns rearrangement, such as operating at mild conditions, without the use of metal catalyst and with reduced organic solvents, affording the desired LacNAc motif with a higher yield and without sub products. Furthermore lactulose is a relatively expensive chemical compared to lactose. A second important example of LacNAc building block application has been provided by Broder et al.,^[28]^ with an more specific investigation of different protective groups. Also in this case the advantages of the enzymatic synthesis we propose compared to this approach are the same as the above mentioned. Enzymatic syntheses of the LacNAc moiety using carbohydrate active enzymes such as galactosyltransferases, glycosynthases and glycosidases have been reported, however lack of selective protection and activation of products has limited applications in automated chemical synthesis.[^29^, ^30^] Herein, we explore the promiscuity of carbohydrate active enzymes that tolerate protecting and activating groups which are needed for subsequent automated chemical glycan assembly (Figure 1).

**Figure 1:**
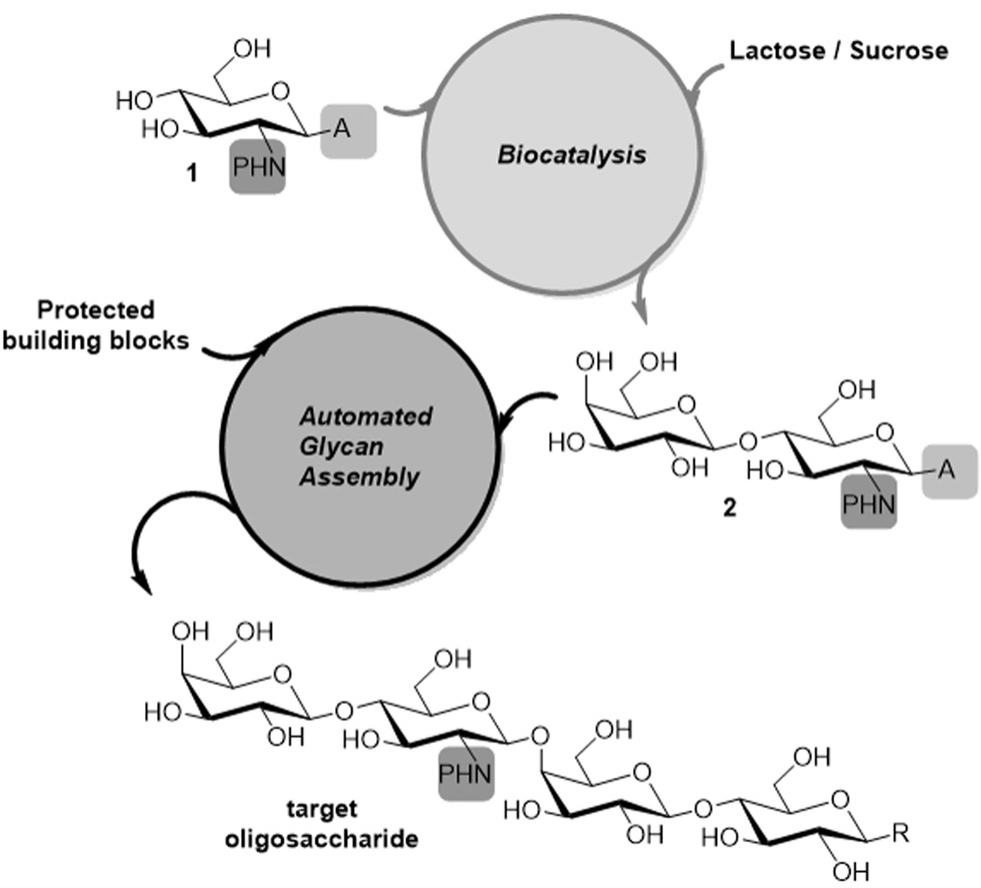
General strategy for combining biocatalysis with AGA. (A=anomeric activating group, for example, S‐Tol; P=activating and β‐directing protecting group, for example, *N*‐trichloroacetyl).

For the LacNAc motif **2**, an activating and β‐directing C2 protecting group (such as *N*‐trichloroacetyl) and anomeric activation as a thioglycoside (such as S‐Tol) are required to ensure selective chemical incorporation of the building block into target tetrasaccharide **3** by automated assembly. This strategy requires an enzyme that can generate **2** from easily available N‐protected glucosamine **1** using cheaply available galactosyl donors, such as lactose or sucrose, rather than expensive sugar nucleotides. We have recently identified and investigated the transgalactosylation activity of NmLgtB‐B,^[31]^ a recombinant bacterial galactosyltransferase (GalT) from *Neisseria meningitidis*. This enzyme has some interesting promiscuous activities and, importantly, it is able to use lactose as an inexpensive galactosyl donor (Figure 2) rather than the expensive UDP‐Gal, thus enabling economically feasible scale‐up. The enzymatic transformation of **1** (reaction Figure 2 a) was carried out in the presence of excess of lactose and UDP to push the reaction towards the formation of the desired product **2**. The product could easily be extracted from the reaction mixture after the removal of the enzyme and evaporation of the reaction media using a mixture of acetonitrile, methanol, ethanol and water (4/2/2/0.5 v/v) followed by chromatographic purification in 83 % yield with robust reproducibility. The starting material could also be easily recovered leading to 97 % of total moles recovered. The purified compound **2** (Figure 2) was quantitatively acetylated with pyridine and acetic anhydride to afford the final building block **3** (Scheme 1) with 82 % of total yield. Interestingly the procedure has been repeated multiple times on different scales, from milligram up to gram scale showing a remarkable reproducibility and linearity in yield. As an alternative towards the formation of the desired compound **2**, we also investigated a one pot three enzyme reaction using sucrose as the starting material.^[32]^ The reaction (Figure 2 b) exploits the reversible action of a glycosyltransferase, in this case sucrose synthase (SuSy), which in presence of substoichiometric quantity of UDP forms UDP‐glucose and fructose (Figure 2). This activated donor can be isomerized by a 4‐glucose epimerase to UDP‐galactose, which can be then transferred by a GalT shifting the equilibrium towards the formation of the desired galactosylated product **2**. Given that both regeneration systems were successful, but path b required three enzymes and path a only one, the latter was chosen for further scale‐up. However, reaction system b may be useful for future application for a multi enzymatic system not compatible with the relatively high amount of lactose and UDP present in the transgalactosylation reaction. In an effort to optimize the unusual transgalactosylation reaction we explored a number of reaction parameters (Figure 3). The pH optimum was found to be pH 7.5 and 15 equivalents of lactose are sufficient to achieve a good conversion. Lastly the concentration of UDP was varied, with a positive result obtained using a concentration of 7.5 mM. The amount of enzymes was found to be a key parameter for a successful reaction with 17.5 % w/w_sm_ the minimum amount required to achieve a conversion of over 80 %. Once robust and reproducible reactions towards the synthesis of product **3** (Scheme 1) were established, we tested it as a building block for automated glycosylation using the Glyconeer 2.1. Synthesis of tetramer **7** was chosen as the model system to display the compatibility of **3** as a starting material in the automated synthesizer (Scheme 2). The reactor was loaded with photocleavable resin (**4**) bearing a hydroxyl group which acted as an acceptor for the first glycosylation cycle using glucoside building block **5**. After the removal of the Fmoc group, exposing the free hydroxyl group in position 4, the second cycle employed galactoside building block **6**. Once again position 4 of the terminal unit was deprotected enabling the glycosylation using the enzymatically prepared building block **3**. The resin was collected and loaded into a UV photoreactor and exposed to UV light to cleave the amino linker, followed by preparative HPLC purification to afford the fully protected compound **7** with an isolated yield of 56 %. In order to obtain good yields every glycosylation was performed using an excess of building blocks **3**, **5** and **6** (two glycosylation reactions using 6.5 equivalents each). The large quantity of starting material for automated synthesis highlights the importance of developing green and economical strategies to access these building blocks.

**Figure 2:**
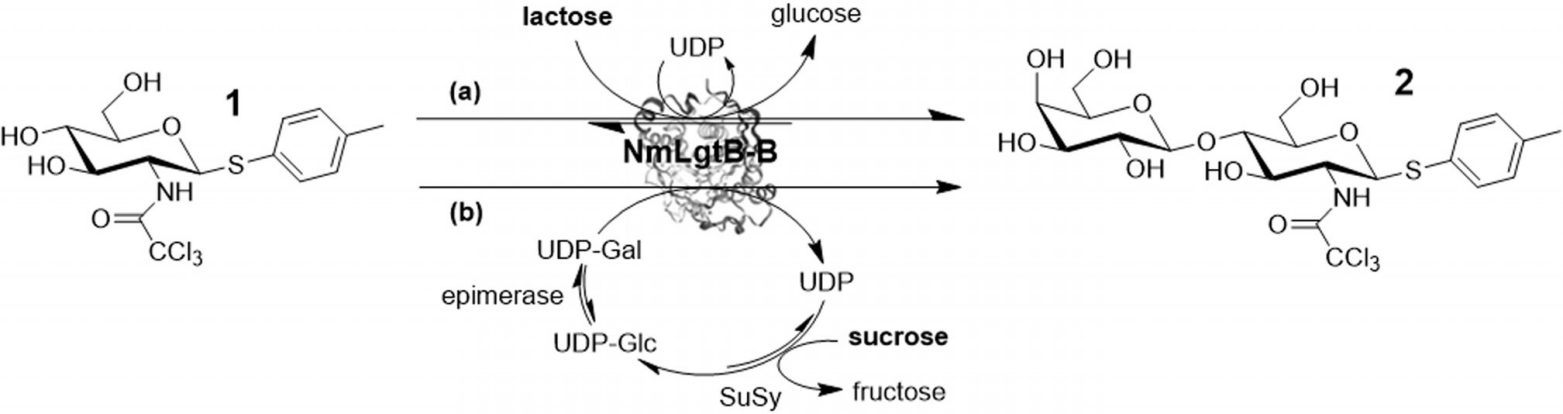
Enzymatic galactosylation of **1** using either lactose (a) or sucrose (b) as inexpensive sources for activated sugar donors.

**Figure 3:**
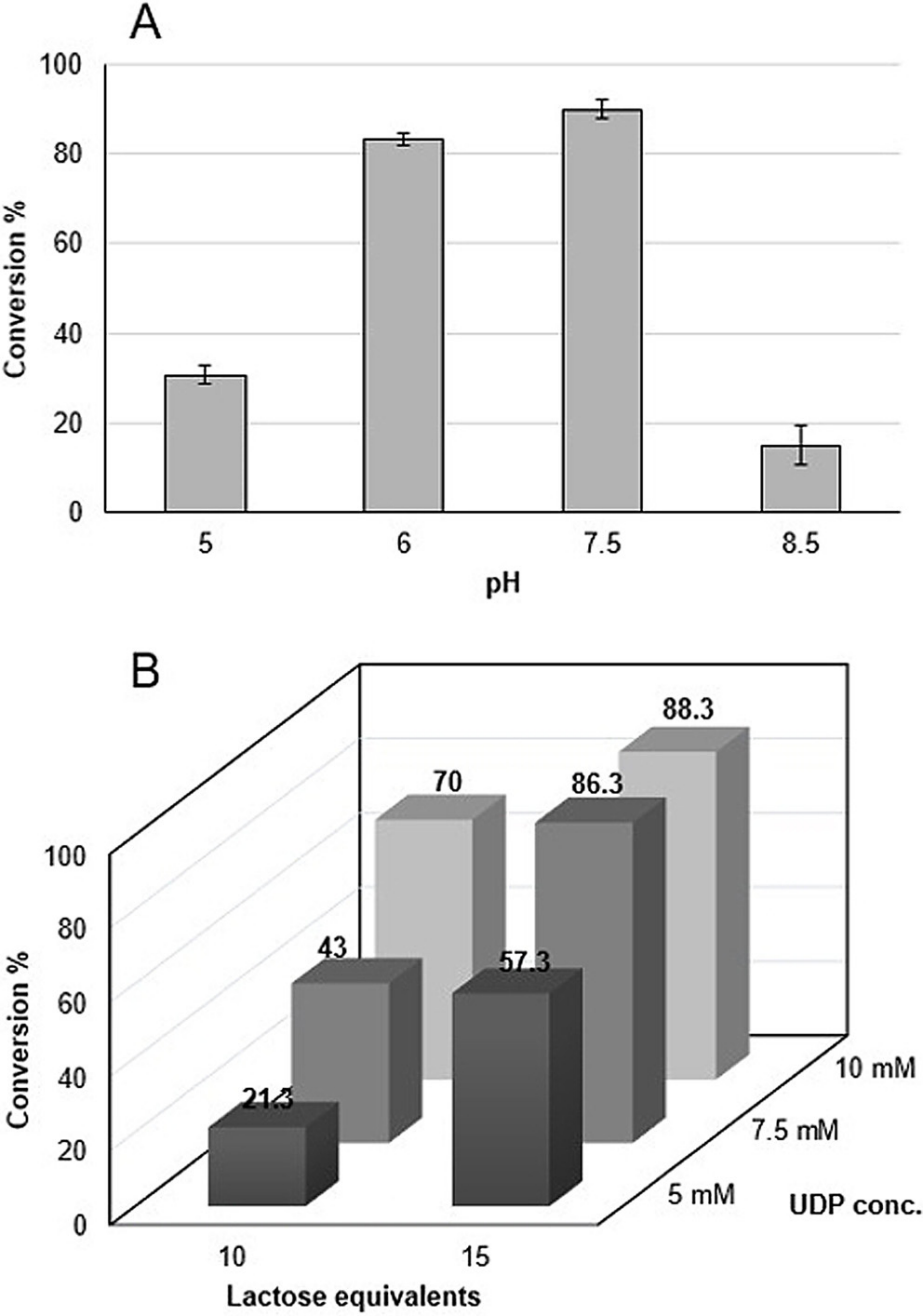
A) Transgalactosylation reaction pH profiling. B) Optimization of the transgalactosylation reaction by varying lactose and UDP equivalents at the optimized pH value.

**Scheme 1:**
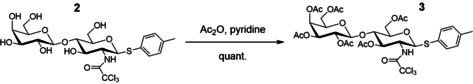
Protection of the enzymatically prepared building block.

**Scheme 2:**
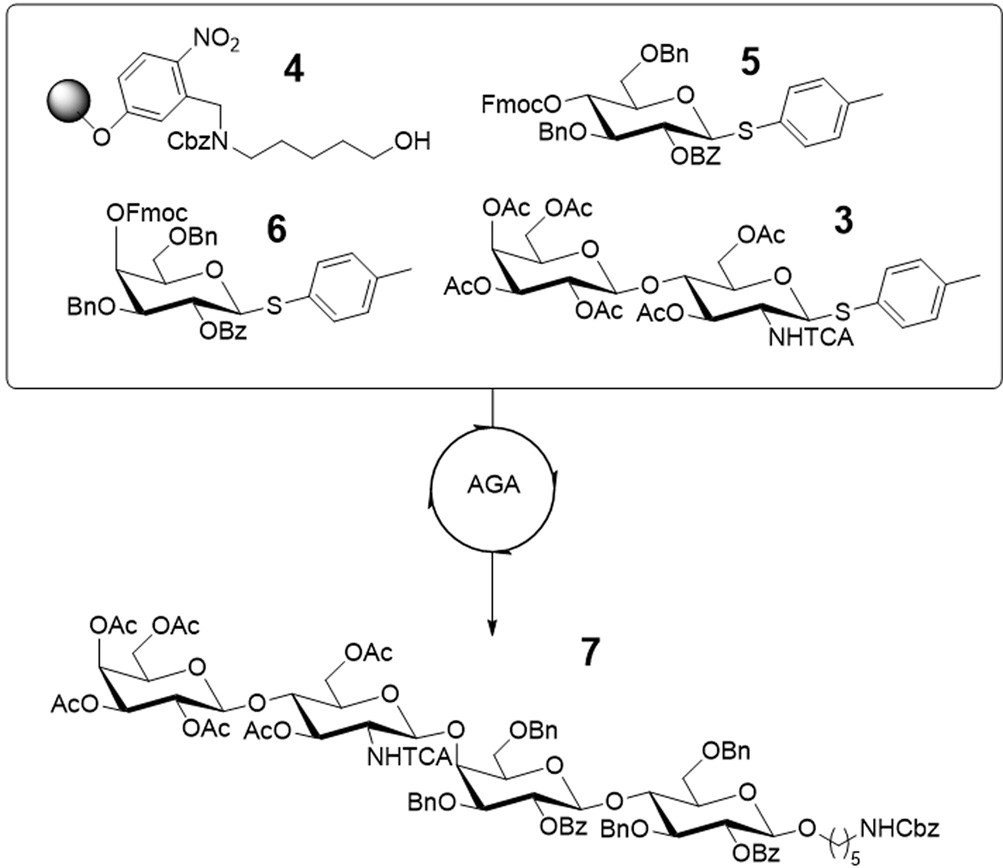
Solid‐phase automated glycan assembly of tetrasaccharide **7** incorporating building block **3**.

In summary, we have demonstrated that selectively protected and activated building blocks can be generated by biocatalysis at gram scale, exploiting the promiscuity of carbohydrate‐active enzymes. The short and efficient synthesis can be scaled up to provide reagents for automated solid‐phase glycan assembly.[^1^, ^3^, ^6^] As microbial glycosyltransferases are becoming more accessible and are increasingly shown to be tolerant to protecting and activating groups, this chemoenzymatic strategy should be a useful addition to modern carbohydrate synthesis.

## Conflict of interest

The authors declare no conflict of interest.

## Supporting information

As a service to our authors and readers, this journal provides supporting information supplied by the authors. Such materials are peer reviewed and may be re‐organized for online delivery, but are not copy‐edited or typeset. Technical support issues arising from supporting information (other than missing files) should be addressed to the authors.

Supplementary

## References

[1] M. Guberman , P. H. Seeberger , J. Am. Chem. Soc. 2019, 141, 5581–5592. 30888803 10.1021/jacs.9b00638 PMC6727384

[2] T. Li , L. Liu , N. Wei , J.-Y. Yang , D. G. Chapla , K. W. Moremen , G.-J. Boons , Nat. Chem. 2019, 11, 229–236. 30792508 10.1038/s41557-019-0219-8 PMC6399472

[3] M. Guberman , M. Brautigam , P. H. Seeberger , Chem. Sci. 2019, 10, 5634. 31293748 10.1039/c9sc00768g PMC6552968

[4] A. Pardo-Vargas , M. Delbianco , P. H. Seeberger , Curr. Opin. Chem. Biol. 2018, 46, 48–55. 29715619 10.1016/j.cbpa.2018.04.007

[5] H. S. Hahm , M. K. Schlegel , M. Hurevich , S. Eller , F. Schuhmacher , J. Hofmann , K. Pagel , P. H. Seeberger , Proc. Natl. Acad. Sci. USA 2017, 114, 3385–3389. 10.1073/pnas.1700141114 PMC5410834 28396442

[6] D. Budhadev , K. Saxby , J. Walton , G. Davies , P. C. Tyler , R. Schworer , M. A. Fascione , Org. Biomol. Chem. 2019, 17, 1817–1821. 30543331 10.1039/c8ob02756k

[7] M. Panza , K. J. Stine , A. V. Demchenko , Chem. Commun. 2020, 56, 1333–1336. 10.1039/c9cc08876h PMC7656230 31930269

[8] F. A. Jaipuri , N. L. Pohl , Org. Biomol. Chem. 2008, 6, 2686–2691. 18633525 10.1039/b803451f

[9] T. Matsushita , I. Nagashima , M. Fumoto , T. Ohta , K. Yamada , H. Shimizu , H. Hinou , K. Naruchi , T. Ito , H. Kondo , S.-I. Nishimura , J. Am. Chem. Soc. 2010, 132, 16651–16656. 21033706 10.1021/ja106955j

[10] N. Bézay , G. Dudziak , A. Liese , H. Kunz , Angew. Chem. Int. Ed. 2001, 40, 2292–2295; 10.1002/1521-3773(20010618)40:12<2292::AID-ANIE2292>3.0.CO;2-D 29711842

[11] P. Sears , C.-H. Wong , Science 2001, 291, 2344–2350. 11269314 10.1126/science.1058899

[12] S. L. Flitsch , Curr. Opin. Chem. Biol. 2000, 4, 619–625. 11102865 10.1016/s1367-5931(00)00152-6

[13] C. Kunz , S. Rudloff , W. Baier , N. Klein , S. Strobel , Annu. Rev. Nutr. 2000, 20, 699. 10940350 10.1146/annurev.nutr.20.1.699

[14] P.-C. Pang , P. C. N. Chiu , C.-L. Lee , L.-Y. Chang , M. Panico , H. R. Morris , S. M. Haslam , K.-H. Khoo , G. F. Clark , W. S. B. Yeung , A. Dell , Science 2011, 333, 1761. 21852454 10.1126/science.1207438

[15] Y. Wang , X. Huang , L.-H. Zhang , X.-S. Ye , Org. Lett. 2004, 6, 4415. 15548039 10.1021/ol0483246

[16] R. Cummings , Mol. BioSyst. 2009, 5, 1087. 19756298 10.1039/b907931a

[17] S. Zhao , I. Walsh , J. L. Abrahams , L. Royle , T. Nguyen-Khuong , D. Spencer , D. L. Fernandes , N. H. Packer , P. M. Rudd , M. P. Campbell , Bioinformatics 2018, 34, 3231–3232. 29897488 10.1093/bioinformatics/bty319

[18] L. Royle , M. P. Campbell , C. M. Radcliffe , D. M. White , D. J. Harvey , J. L. Abrahams , Y.-G. Kim , G. W. Henry , N. A. Shadick , M. E. Weinblatt , D. M. Lee , P. M. Rudd , R. A. Dwek , Anal. Biochem. 2008, 376, 1–12. 18194658 10.1016/j.ab.2007.12.012

[19] S. Honda , S. Suzuki , A. Nitta , S. Iwase , K. Kakehi , Methods 1992, 4, 233–243.

[20] S. Suzuki , K. Kakehi , S. Honda , Anal. Biochem. 1992, 205, 227–236. 1443568 10.1016/0003-2697(92)90428-a

[21] H. Paulsen , K.-M. Steiger , Carbohydr. Res. 1987, 169, 105–125.

[22] T. Fischöder , D. Laaf , C. Dey , L. Elling , Molecules 2017, 22, 1320. 10.3390/molecules22081320 PMC6152129 28796164

[23] W. Peng , J. Pranskevich , C. Nycholat , M. Gilbert , W. Wakarchuk , J. C. Paulson , N. Razi , Glycobiology 2012, 22, 1453–1464. 22786570 10.1093/glycob/cws101 PMC3481905

[24] Z. Xiao , Y. Guo , Y. Liu , L. li , Q. Zhang , L. Wen , X. Wang , S. M. Kandengaden , Z. Wu , J. Zhou , X. Cao , X. Li , C. Ma , P. G. Wang , J. Org. Chem. 2016, 81, 5851–5865. 27305319 10.1021/acs.joc.6b00478 PMC5953189

[25] J. Xue , S. D. Khaja , R. D. Locke , K. L. Matta , Synlett 2004, 0861–0865.

[26] P. Peng , H. Liu , J. Gong , J. M. Nicholls , X. Li , Chem. Sci. 2014, 5, 3634–3639.

[27] T. M. Wrodnigg , A. E. Stütz , Angew. Chem. Int. Ed. 1999, 38, 827–828; 10.1002/(SICI)1521-3773(19990315)38:6<827::AID-ANIE827>3.0.CO;2-N 29711798

[28] W. Bröder , H. Kunz , Bioorg. Med. Chem. 1997, 5, 1–19. 9043654 10.1016/s0968-0896(96)00209-x

[29] H. Cao , S. Huang , J. Cheng , Y. Li , S. Muthana , B. Son , X. Chen , Carbohydr. Res. 2008, 343, 2863–2869. 18639240 10.1016/j.carres.2008.06.020 PMC2783551

[30] D. Schmidt , J. Thiem , Beilstein J. Org. Chem. 2010, 6, 18. 20485600 10.3762/bjoc.6.18 PMC2871369

[31] K. Huang , F. Parmeggiani , H. Ledru , K. Hollingsworth , J. M. Pons , A. Marchesi , P. Both , A. P. Mattey , E. Pallister , G. S. Bulmer , J. M. van Munster , W. B. Turnbull , M. C. Galan , S. L. Flitsch , Org. Biomol. Chem. 2019, 17, 5920–5924. 31165848 10.1039/c9ob01089k

[32] C. H. Hokke , A. Zervosen , L. Elling , D. H. Joziasse , V. H. Van Den Eijnden , Glycoconjugate J. 1996, 13, 687–692. 10.1007/BF00731458 8872127

