# Supplementary material for: Enzymatic Building‐Block Synthesis for Solid‐Phase Automated Glycan Assembly
Source: Angew Chem Int Ed Engl. 2020 Oct 2;59(50):22456–9. doi: 10.1002/anie.202008067 (PMC7756758; doi:10.1002/anie.202008067)
Supplement: Supplementary file 1 — Supplementary [file ANIE-59-22456-s001.pdf]

Angewandte Chemie
Eine Zeitschrift der Gesellschaft Deutscher Chemiker

Supporting Information

Enzymatic Building-Block Synthesis for Solid-Phase Automated Glycan Assembly

*Andrea Marchesi, Fabio Parmeggiani, João Louçano, Ashley P. Mattey, Kun Huang, Tanistha Gupta, Mario Salwiczek, and Sabine L. Flitsch**

anie_202008067_sm_miscellaneous_information.pdf

SUPPORTING INFORMATION

Table of contents

General methods and materials page S2

Chemo-enzymatic synthesis of N-acetyl-lactosamine building block page S3

Automated glycan assembly using Glyconeer page S6

Analytical data page S8

References page S16

General methods and materials

All reagents were acquired from commercial sources unless stated otherwise. The enzymes UDP-glucose 4-epimerase and NmLgtB-B has been provided by Prozomix Ltd. as suspension of purified protein in 3.2 M ammonium sulphate, SlSuSy has been expressed in *E. coli* cells following a standard protocol for transformation and expression in *E. coli* BL21(DE3) cells.

^{1}H and ^{13}C NMR were recorded on a Bruker Avance 400 instrument (400 MHz for ^{1}H and ^{13}C) in $CDCl_3$ or CD_3OD using residual protic solvent as an internal standard. Reported chemical shifts (δ) in parts per million (ppm) are relative to the residual protic solvent signal.

Reactions were monitored by TLC using silica gel 60 F254 TLC plates (EtOAc / EtOH 6/4) with analysis at UV light (254 nm) and after staining using *p*-Anisaldehyde staining. The conversion rate was determined using a reverse-phase HPLC analysis performed on an Agilent 1200 series LC system equipped with a Luna C18 250 × 2 mm reverse phase column, according to the following method. Mobile phase A: 50 mM ammonium formate in water, pH 4.5. Mobile phase B: acetonitrile. Flow rate: 0.6 mL/min. Gradient: 0-5 min isocratic 5% B, 5-15 min linear gradient 5-50% B, 15-25 min isocratic 5% B. Preparative HPLC used the same method as before using a HyperClone 5u ODS (C18) 250 x 21.20 mm column.

__Chemo-enzymatic synthesis of *N*-acetyl-lactosamine building block__

Enzymatic synthesis of compound 2 with sucrose

GlcNTCA-STol (5 mM) was dissolved in a 50 µL reaction mixture containing sucrose (50 mM), UDP (0.5 mM), $MnCl_2$ (5 mM), $MgCl_2$ (5 mM), Tris buffer pH 6.0 (50 mM), UDP-glucose 4-epimerase (0.12 mg), NmLgtB (0.05 mg) and SlSuSy (0.02 mg). The reaction mixture was incubated at 37°C for 24 h and then filtered using 0.2 mL Vivaspin columns (30kDa MWCO) to remove the enzymes. The solution was evaporated under vacuum and resuspended in 30 µL of water/acetonitrile 95/5 and sonicated. The formation of the product was confirmed by MALDI mass spectrometry and quantified by HPLC using a column C18 Phenomenex using a gradient from 5% to 80% acetonitrile over 30 min, with a conversion of 93%.

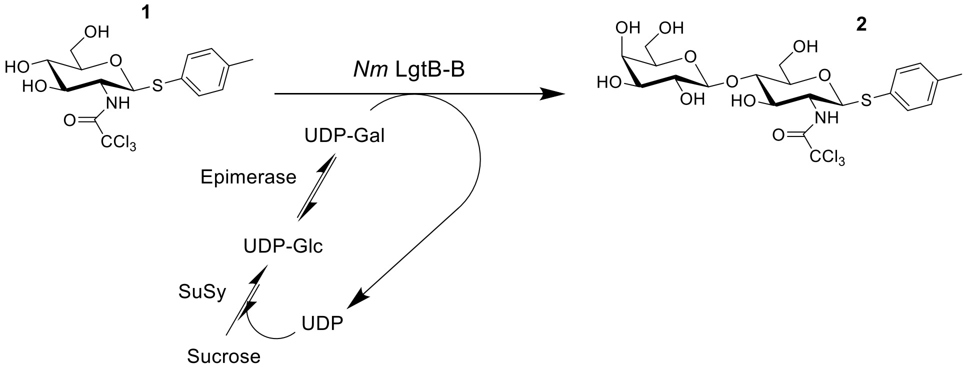

Scheme S1: synthesis of compound **2** starting using sucrose.

Enzymatic synthesis of compound 2 with lactose

GlcNTCA-STol (5 mM) was dissolved in a 50 uL reaction mixture containing lactose (75 mM), UDP (10 mM), $MnCl_2$ (5 mM), $MgCl_2$ (5 mM), Tris buffer pH 7.5 (50 mM) and NmLgtB (0.05 mg). The reaction mixture was incubated at 37°C for 24 h and then filtered using 0.2 mL Vivaspin columns (30kDa MWCO) to remove the enzymes. The solution was evaporated under vacuum and resuspended in 30 µL of water/acetonitrile 95/5 and sonicated. The formation of the product was confirmed by MALDI mass spectrometry and quantified by HPLC using a column C18 Phenomenex using a gradient from 5% to 80% acetonitrile over 30 min, with a conversion of 94%.

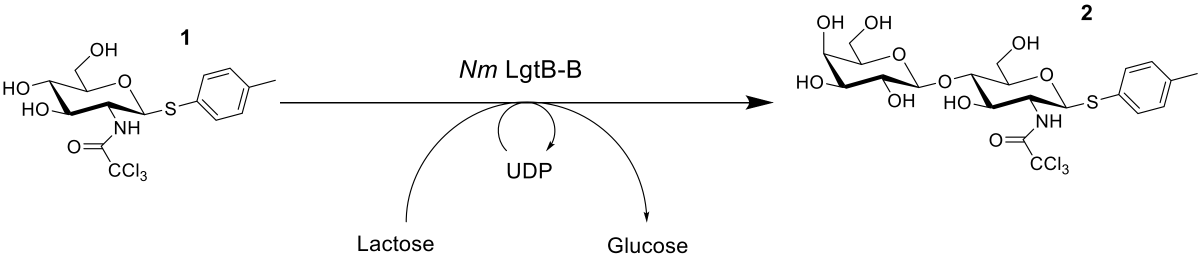

Scheme S2: synthesis of compound **2** using lactose.

Preparative enzymatic synthesis of compound 2

GlcNTCA-STol (200 mg, 0.465 mmol) was dissolved in DMSO (2.325 mL) and water (2.325 mL) and was added to a 93 mL reaction mixture containing UDP (416 mg, 0.93 mmol), lactose monohydrate (2546 mg, 6.975 mmol), $MnCl_2$ (5 mM), $MgCl_2$ (5 mM), Tris buffer pH 7.5 (50 mM) and NmLgtB (75 mg). The reaction mixture was incubated at 37°C in a shaking incubator (220 rpm) for 18 h. The reaction mixture was then centrifuged at 4000 rpm and room temperature to remove the enzyme precipitated. The supernatant was filtered using 20 mL Vivaspin columns (30kDa MWCO) to remove the soluble portion of the enzyme. The solution was evaporated under vacuum and the resulting solid was suspended in acetonitrile (4 mL), ethanol (4 mL), methanol (2 mL) and water (0.5 mL) vortexing for 10 minutes and sonicating for 10 min. The resulting suspension was centrifuged to separate a large excess of the insoluble lactose from the supernatant containing the product. This procedure was repeated a second time on the insoluble fraction and the supernatants were pooled and evaporated under vacuum. The residue was then suspended in water and acetonitrile 95/5 and the product was purified by preparative HPLC C18 Phenomenex column, using a gradient from 5% to 80% acetonitrile over 30 minutes. The fractions containing the product were evaporated under vacuum and the solid residue suspended in water and freeze dried to obtain the product as white powder in 85% yield.

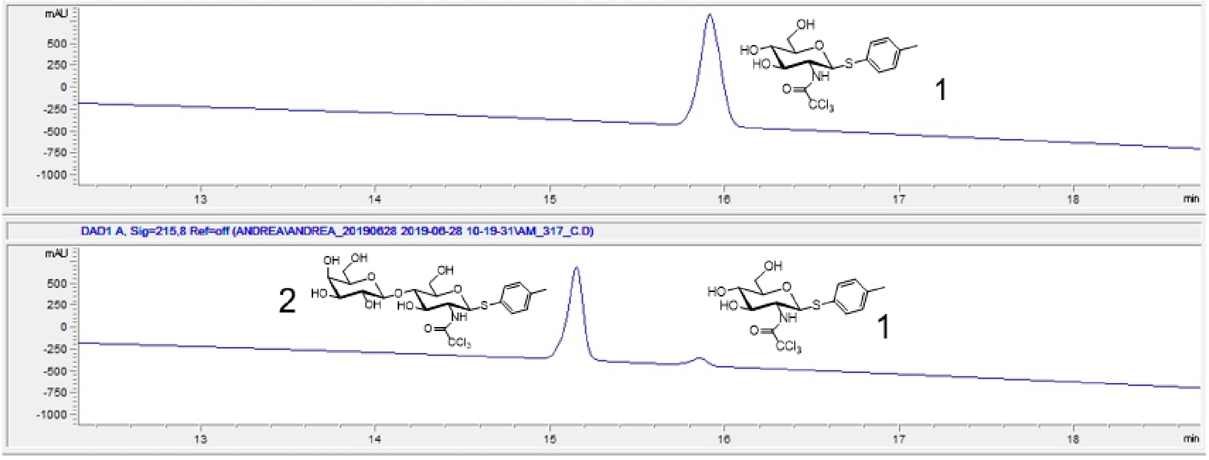

Figure S3: HPLC traces of starting material **1** and product **2**.

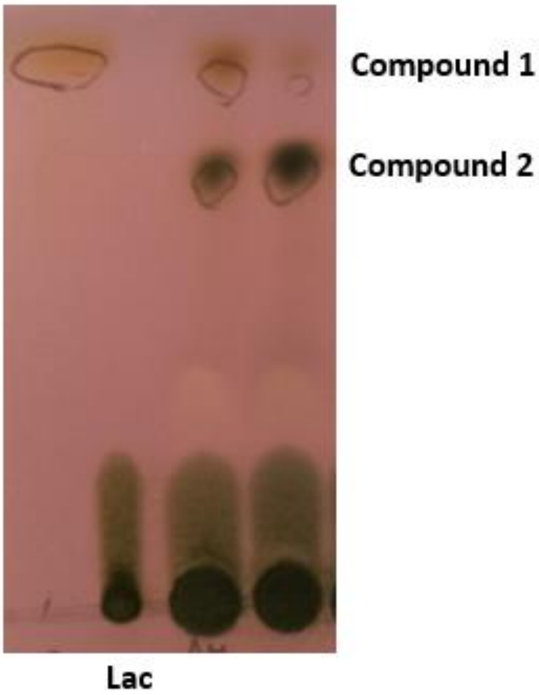

Figure S4: TLC analysis performed in eluent EtOAc/EtOH 6/4.

Protection of compound 2 (synthesis of building block 3)

LacNTCA-STol (500 mg, 0.905 mmol) was dissolved in pyridine (10 mL) and the reaction mixture was cooled at 0°C using an ice bath. Ac_2O (15 mL) was then added to the reaction mixture; the reaction was allowed to warm at room temperature and was left to stir for 24 h. The reaction was then diluted with ethyl acetate (20 mL) and transferred to a separation funnel. The organic phase was washed with a saturated solution of $NaHCO_3$ (2x 40 mL), water (20 mL), HCl 5% solution (2x 40 mL) and brine (20 mL). The organic phase was then dried over anhydrous $MgSO_4$, filtered and evaporated under vacuum, giving the product as a colourless viscous oil in quantitative yield.

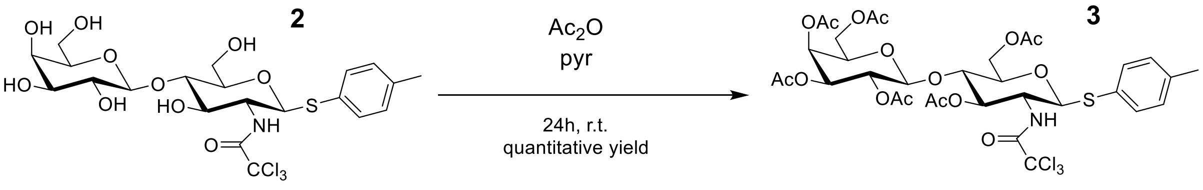

Scheme S5: Protection reaction to achieve building block compound **3**.

Automated glycan assembly using Glyconeer

All solvents were loaded in the respective bottle and attached in the corresponding position of the Glyconeer prior to the synthesis. For each synthesis, the building blocks were co-evaporated with toluene, dried under high vacuum for 2 h, dissolved in anhydrous DCM (1 mL) and transferred to oven-dried building block vials. The vials were then placed in the appropriate position in the building block carousel. The reagent solutions were prepared as described below. A polystyrene base resin functionalized with 0.40 mmol/g of a photocleavable linker was used. The resin (25 µmol of hydroxyl groups, 31 mg of resin) was weighed and transferred into the reaction vessel and the synthesis programme was initiated.

Preparation of reagent solutions:

TMSOTf solution: anhydrous DCM (40 mL) was transferred into an oven-dried reagent bottle under Ar. TMSOTf (0.45 mL) was added and the bottle was placed in the predetermined acid-wash position and attached to the Glyconeer.

Activation solution: *N*-iodosuccinimide (NIS, 1.35 g) was added to an oven-dried reagent bottle. Anhydrous dioxane (13 mL) and anhydrous DCM (26 mL) were added and the mixture gently stirred. Triflic acid (TfOH, 55 µL) was added and the bottle placed in the cooling block, in the predetermined position, and attached to the Glyconeer.

Pyridine solution: pyridine (5 mL) was diluted with DMF (45 mL) and the resulting solution was transferred into a reagent bottle. The bottle was placed in the predetermined position and attached to the Glyconeer.

Capping solution: anhydrous DCM (36 mL) was transferred into an oven-dried reagent bottle under Ar. Ac_2O (4 mL) was added followed by MsOH (0.8 mL). The bottle was placed in the predetermined position and attached to the Glyconeer.

Piperidine solution: piperidine (50 mL) was diluted with DMF (200 mL) and the resulting solution was transferred into a reagent bottle. The bottle was placed in the predetermined position and attached to the Glyconeer.

All experiments in the Glyconeer start with a “Resin swelling” module. During this module, DCM (2 mL) is delivered in the reaction vessel and the resin is incubated at 25°C for 30 min. During the incubation time, the machine rinses the manifolds. Afterwards, syntheses were programmed by combining the pre-defined modules described below. For each monosaccharide to be added to the target glycan, the cycle Acid Wash, Glycosylation, Capping, Fmoc Deprotection was repeated.

Module 1 – Acid Wash: the temperature is set to –20°C and the resin is washed with the TMSOTf solution (1 mL).

Module 2 – Glycosylation: The temperature is adjusted to the addition temperature T1 (–20°C unless otherwise stated), the building block solution (1 mL) is delivered and then the activator solution (1 mL). An incubation period t1 = 5 min is followed. The temperature is then adjusted to T2 (0°C unless otherwise stated) and an incubation period t2 = 20 min is followed. The resin is then washed with a 1:1 solution of DCM/dioxane, and then with DCM. When a double coupling was required, the

procedure was repeated. When a quadruple coupling was required, the procedure was repeated to a total of four times.

Module 3 – Capping: The temperature is set to 25 °C. The resin is washed with DMF, then with the pyridine solution (2 mL), then with DCM. The capping solution is added (4 mL) and resin is incubated for 20 min. The resin is finally washed with DCM.

Module 4 – Fmoc Deprotection: The temperature is set to 25 °C. The resin is washed with DMF and then the piperidine solution (2 mL) is added. The resin is incubated for 5 min and afterwards washed with DMF and DCM.

General procedure for cleavage from the solid support and purification

After automated synthesis in the Glyconeer, the solid support was suspended in DCM and injected into a continuous-flow photoreactor as described previously[S1] to release the generated oligosaccharides. The resulting crude material was analysed by analytical HPLC using a YMC-Diol-300 column (150 x 4.6 mm) and the following elution method: flow rate 1 mL/min, elution started with 20% EtOAc in hexane for 5 min (isocratic), then linear gradient to 55% EtOAc in hexane for 35 min, then linear gradient to 100% EtOAc for 5 min, then 100% EtOAc for 5 min (isocratic). The target oligosaccharide was purified using a preparative HPLC using a YMC-Diol-300 column (150 x 20 mm) and the following elution method: flow rate 15 mL/min, elution started with 20% EtOAc in hexane for 5 min (isocratic), then linear gradient to 55% EtOAc in hexane for 35 min, then linear gradient to 100% EtOAc for 5 min, then 100% EtOAc for 5 min (isocratic). Fractions were collected, evaporated under reduced pressure and dried under high vacuum overnight affording compound **7** in 56% yield (25.8 mg) calculated starting from the µmol of hydroxyl groups on the resin.

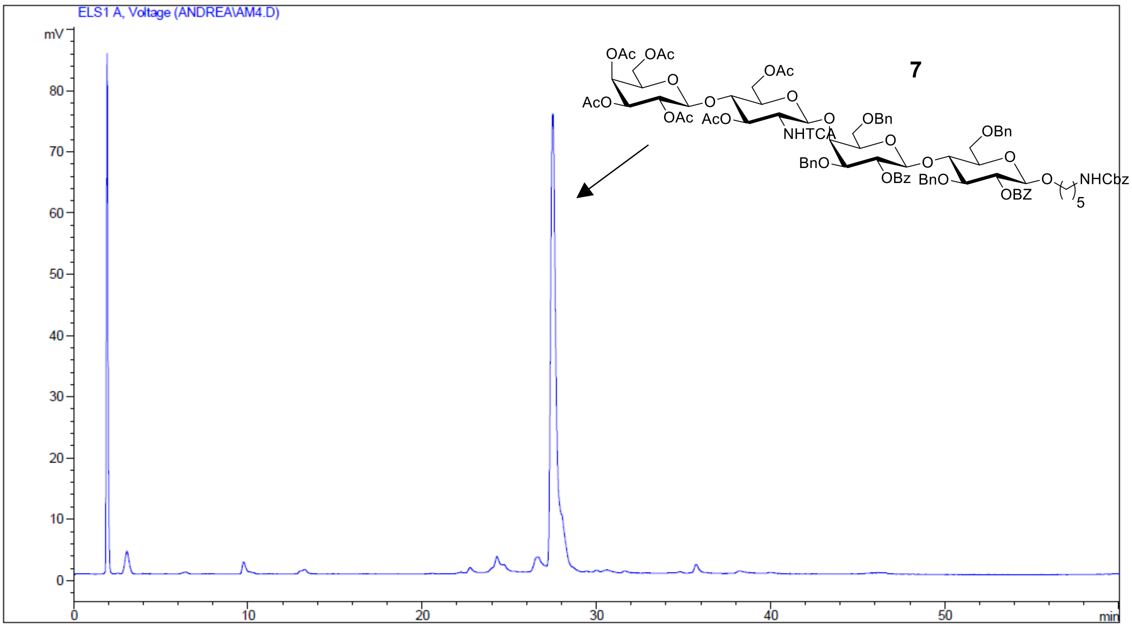

Figure S6: preparative HPLC trace for the purification of compound **7**.

Compound analytical data

Compound 2

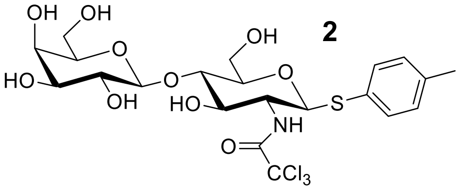

2

^{1}H NMR (400 MHz, CD_3OD) δ 7.40 (d, *J* = 8.2 Hz, 2H), 7.11 (d, *J* = 8 Hz, 2H), 4.37 (d, *J* = 7.1 Hz, 1H), 3.92 (dd, *J* = 12.3, 2.5 Hz, 1H), 3.85 (dd, *J* = 12.3, 4.3 Hz, 1H), 3.81-3.72 (m, 4H), 3.64 (ddd, *J* = 15.7, 8.9, 3.6 Hz, 2H), 3.60-3.53 (m, 1H), 3.53-3.45 (m, 2H), 3.42 (ddd, *J* = 9.8, 4.3, 2.5 Hz, 1H), 2.29 (s, 3H).

^{13}C NMR (400 MHz, CD_3OD) δ 162.51, 137.70, 132.29, 129.80, 129.23, 103.63, 86.83, 79.34, 79.25, 75.79, 73.66, 73.43, 71.27, 69.00, 61.29, 60.60, 56.04, 19.67

Expected mass 591.05 found 613.764 = M+[Na^{+}- H^{+}]

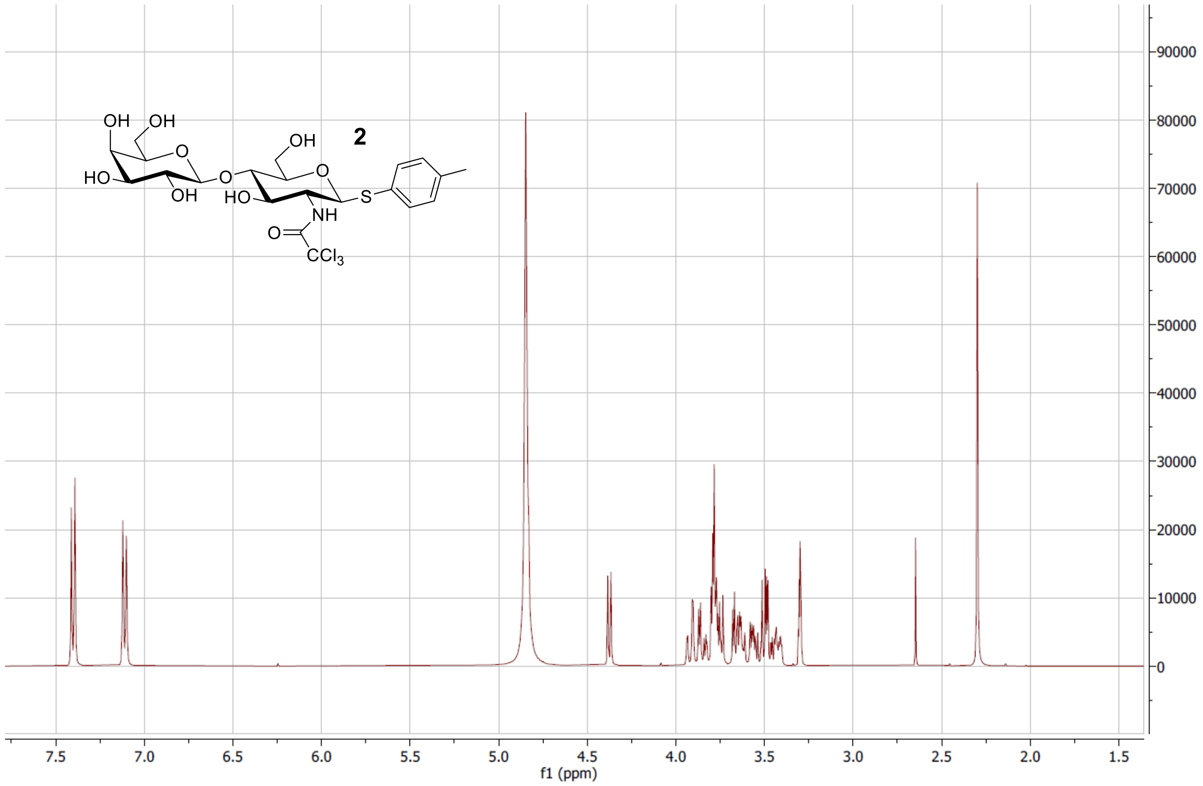

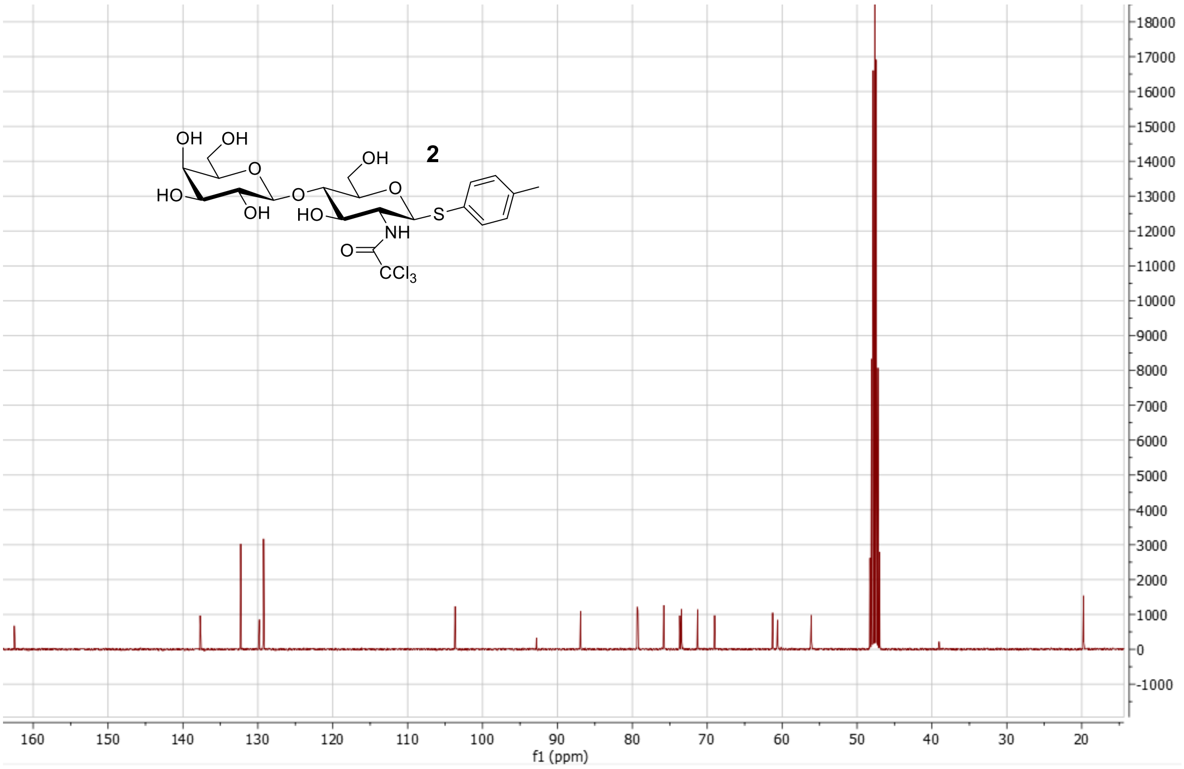
2
f1 (ppm)
160
150
140
130
120
110
100
90
80
70
60
50
40
30
20
18000
17000
16000
15000
14000
13000
12000
11000
10000
9000
8000
7000
6000
5000
4000
3000
2000
1000
0
-1000

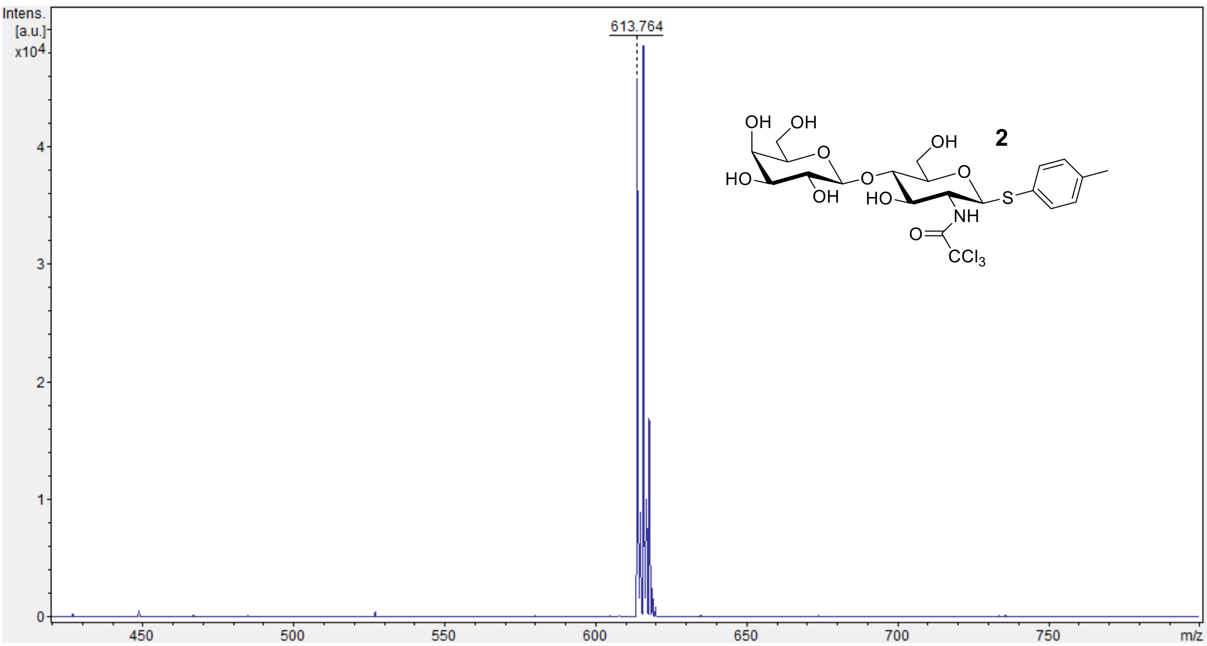
Intens.
[a.u.]
x10^4
613.764
2
m/z
450
500
550
600
650
700
750
0
1
2
3
4

Compound **3**

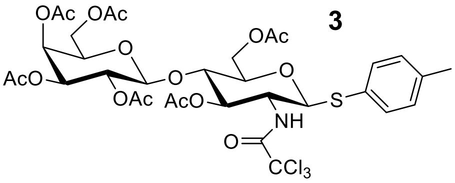

3

^{1}H NMR (400 MHz, $CDCl_3$) δ 7.30 (d, *J* = 8.1 Hz, 2H, aromatic ring), 7.10 (d, *J* = 3.6 Hz, 1H, -N*H*), 7.02 (d, *J* = 7.9 Hz, 2H, aromatic ring), 5.27 (d, *J* = 2.9 Hz, 1H, *H*4'), 5.16 (dd, *J* = 10.2, 8.8 Hz, 1H, *H*3), 5.01 (m, 1H, *H*2'), 4.88 (dd, *J* = 10.4, 3.4 Hz, 1H, *H*3'), 4.63 (d, *J* = 10.33 Hz, 1H, *H*1), 4.51 (dd, *J* = 11.9, 2.1 Hz, 1H, *H*6), 4.42 (d, *J* = 7.9 Hz, 1H, *H*1'), 4.01 (m, 3H, *H*6 + *H*6'), 3.91 (q, *J* = 10 Hz, 1H, *H*2), 3.80 (t, *J* = 6.6 Hz, 1H, *H*5'), 3.69 (t, *J* = 9.3 Hz, 1H, *H*4), 3.6 (m, 1H, *H*5), 2.26 (s, 3H, -CH_3), 2.06-2.05 (s, 6H, [2x] -COCH_3), 1.97-1.96 (s, 9H, [3x] -COCH_3), 1.89 (s, 3H, -COCH_3).

^{13}C NMR (400 MHz, $CDCl_3$) δ 170.70, 170.35, 170.18, 170.08, 169.18, 161.75, 138.72, 133.81, 129.66, 127.41, 101.36, 92.28, 86.03, 77.38, 77.06, 76.74, 76.32, 73.53, 70.94, 70.70, 69.08, 66.57, 62.07, 60.77, 54.32, 30.94, 21.18, 20.88, 20.78, 20.62, 20.51

Expected mass 843.11 found 865.851 = M+[Na^+] and 882.142 = M+[K^+]

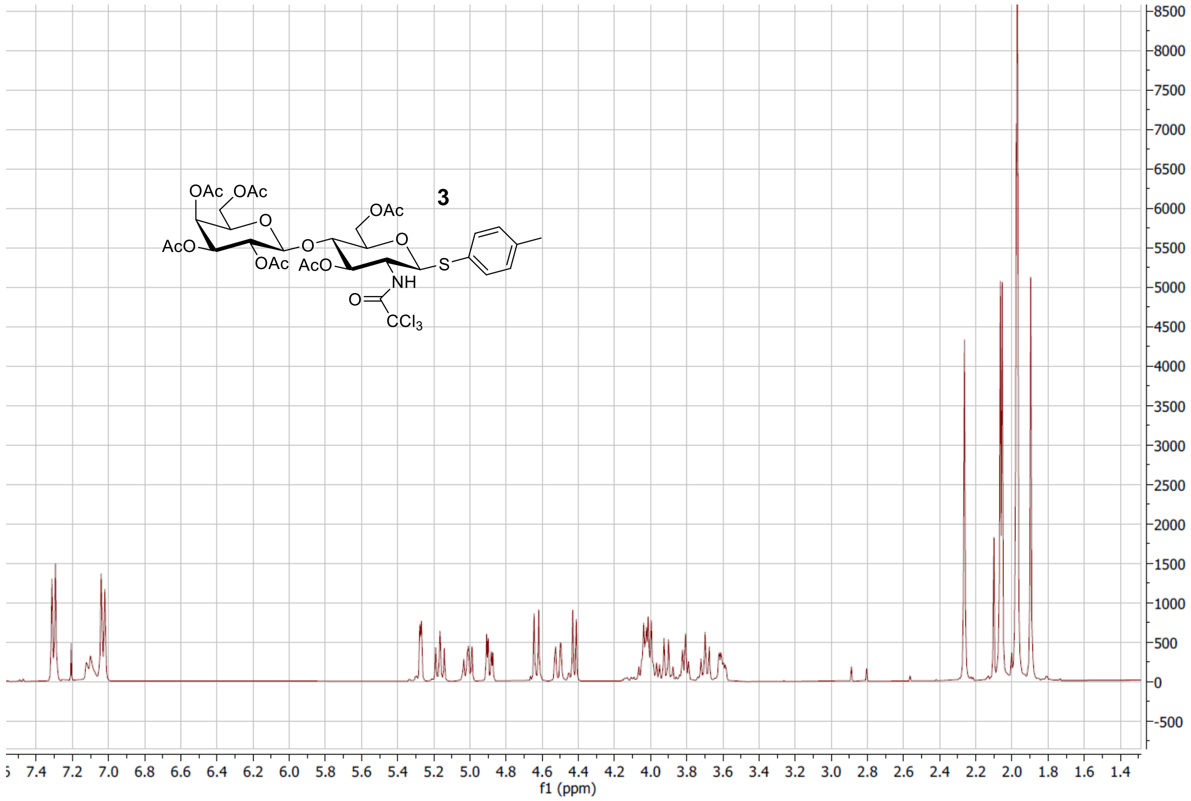

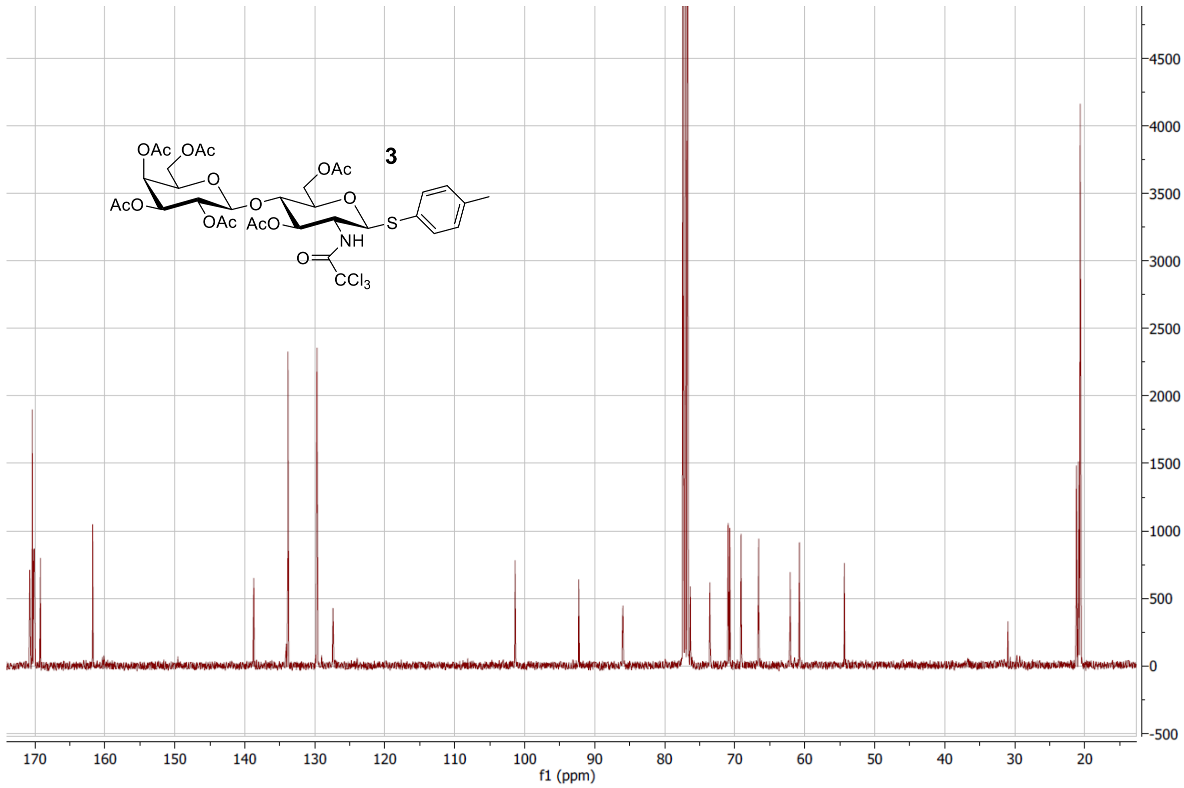

3
OAc OAc
AcO
OAc
OAc
AcO
NH
O
S
CCl3
f1 (ppm)

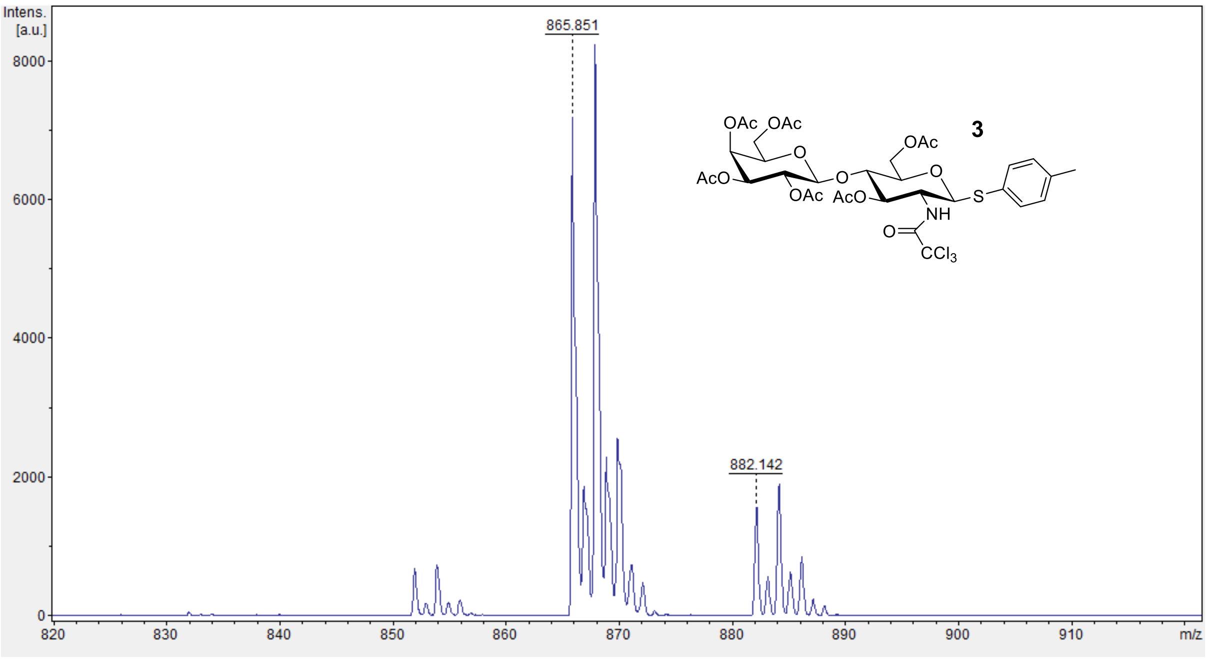

Intens. [a.u.]
865.851
882.142
3
m/z

Compound **7**

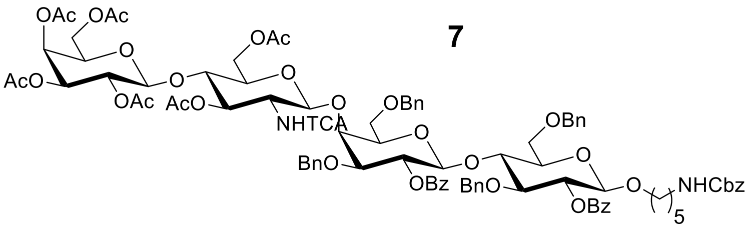

^{1}H NMR (400 MHz, $CDCl_3$) δ 7.86 (m, 5H), 7.54-6.94 (m, 35H), 6.99 (d, 1H), 5.26 (m, 2H), 5.11-4.97 (m, 4H), 4.94 (d, 1H), 4.88 (dd, 1H), 4.81 (d, 1H), 4.56 (d, 1H), 4.52-4.37 (m, 6H), 4.26 (m, 3H), 4.19 (m, 2H), 4.02 (dd, 2H), 3.97 (m, 1H), 3.90 (q, 2H), 3.77 (m, 2H), 3.64 (q, 2H), 3.50 (m, 2H), 3.45 (d, 1H) 3.38 (m, 2H), 3.29 (m, 1H), 3.20 (m, 2H), 2.79 (m, 2H), 2.07 (s, 3H), 1.97-1.96 (s, 12H), 1.89 (s, 3H), 1.35 (m, 2H), 1.18 (m, 4H), 1.05 (m, 2H).

^{13}C NMR (500 MHz, $CDCl_3$) δ 170.37, 170.21, 170.15, 170.08, 169.09, 165.08, 164.80, 162.28, 156.23, 138.64, 138.38, 138.27, 137.00, 136.73, 133.32, 132.96, 130.07, 129.77, 129.66, 128.80, 128.56, 128.49, 128.44, 128.38, 128.36, 128.34, 128.09, 128.04, 128.02, 127.90, 127.86, 127.78, 127.66, 127.58, 127.50, 126.95, 101.29, 101.08, 100.85, 99.73, 92.14, 80.66, 79.66, 76.25, 74.82, 74.67, 73.57, 73.38, 73.32, 73.11, 72.71, 72.61, 72.44, 71.16, 70.96, 70.65, 69.35, 69.15, 68.69, 67.90, 66.54, 66.45, 62.15, 61.87, 60.73, 55.81, 40.78, 29.34, 28.82, 23.01, 20.78, 20.66, 20.53.

Expected mass 1848.56 found 1871.607 = M+[Na^{+}]

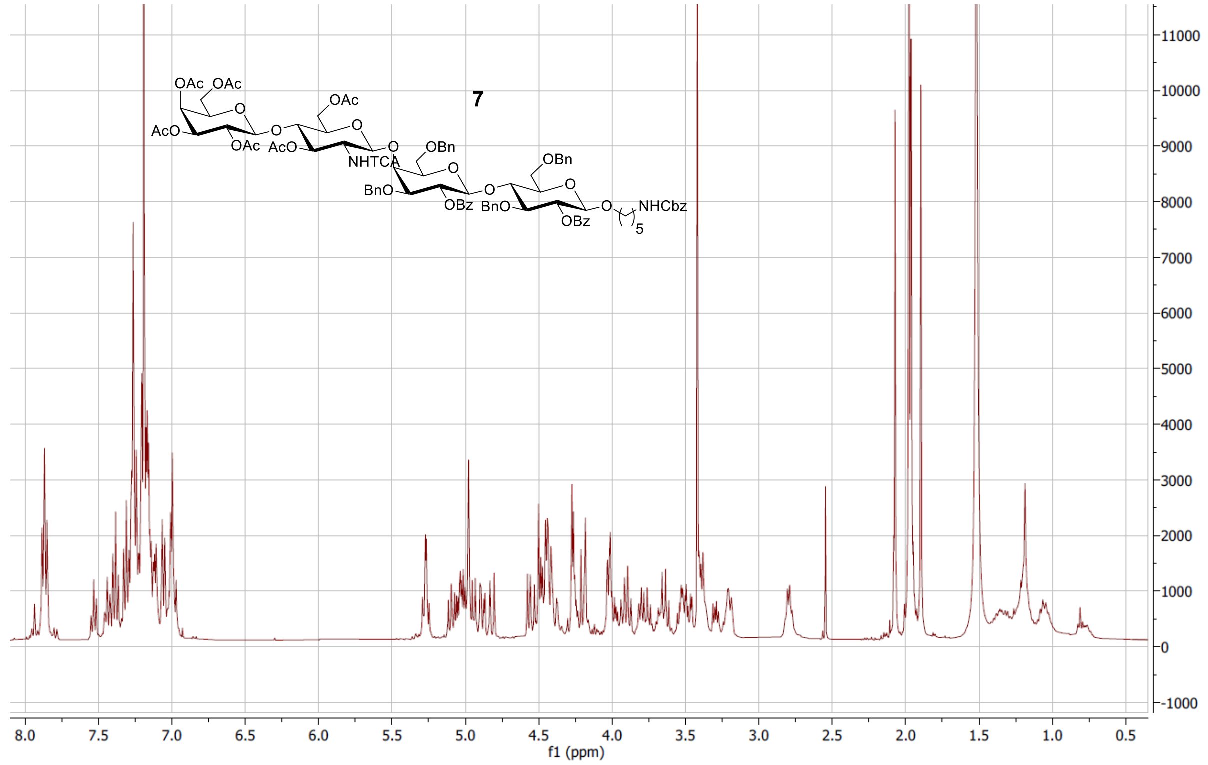

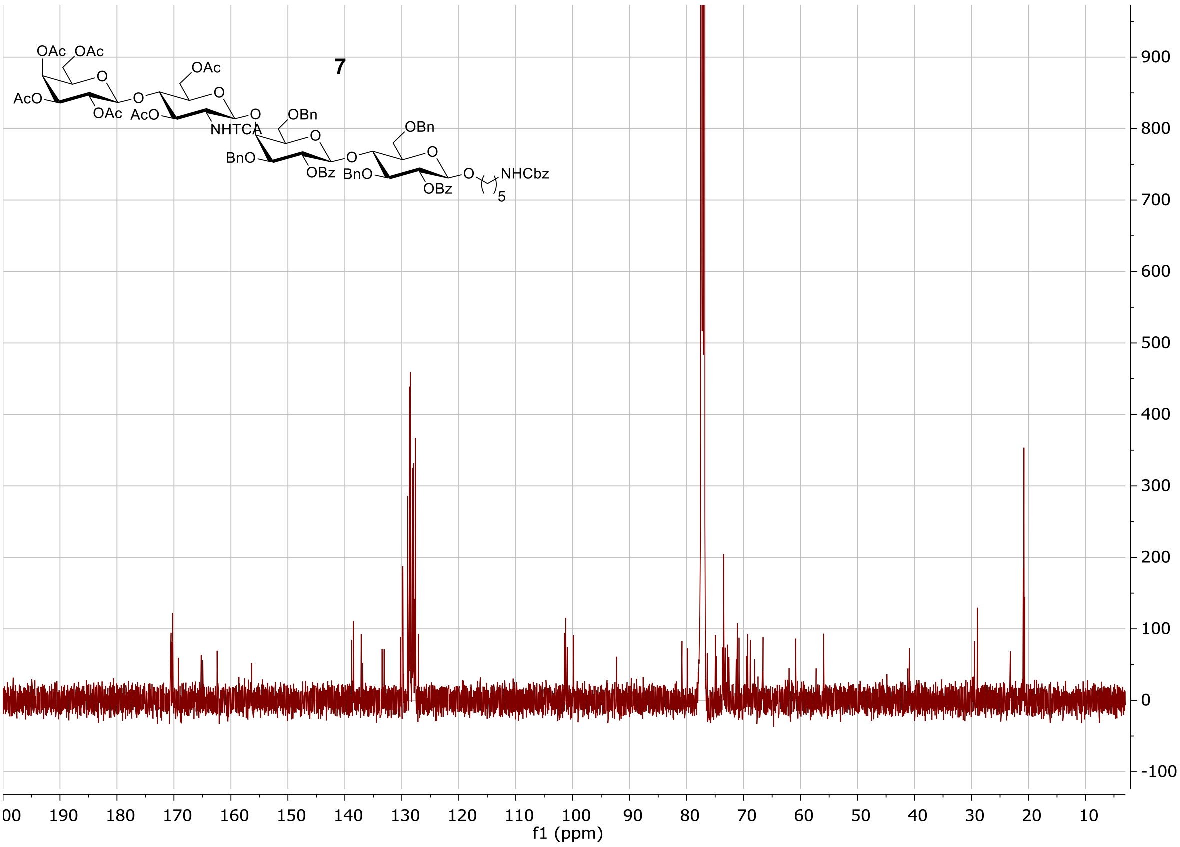
7
OAc OAc
AcO
OAc AcO
OAc
NHTCA
OBn
BnO
OBz BnO
OBn
OBz
NHCbz
5
f1 (ppm)

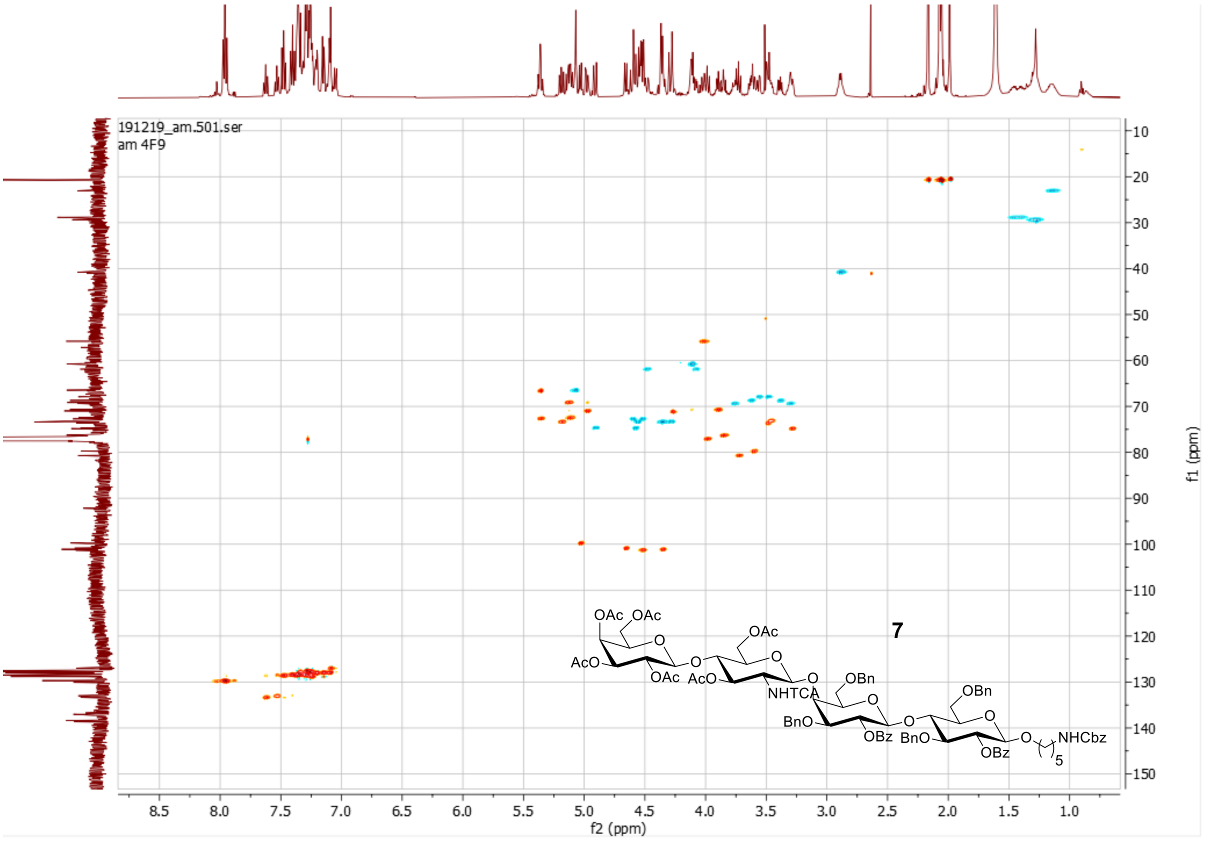
191219_am.501.ser
am 4F9
7
OAc OAc
AcO
OAc AcO
OAc
NHTCA
OBn
BnO
OBz BnO
OBn
OBz
NHCbz
5
f2 (ppm)
f1 (ppm)

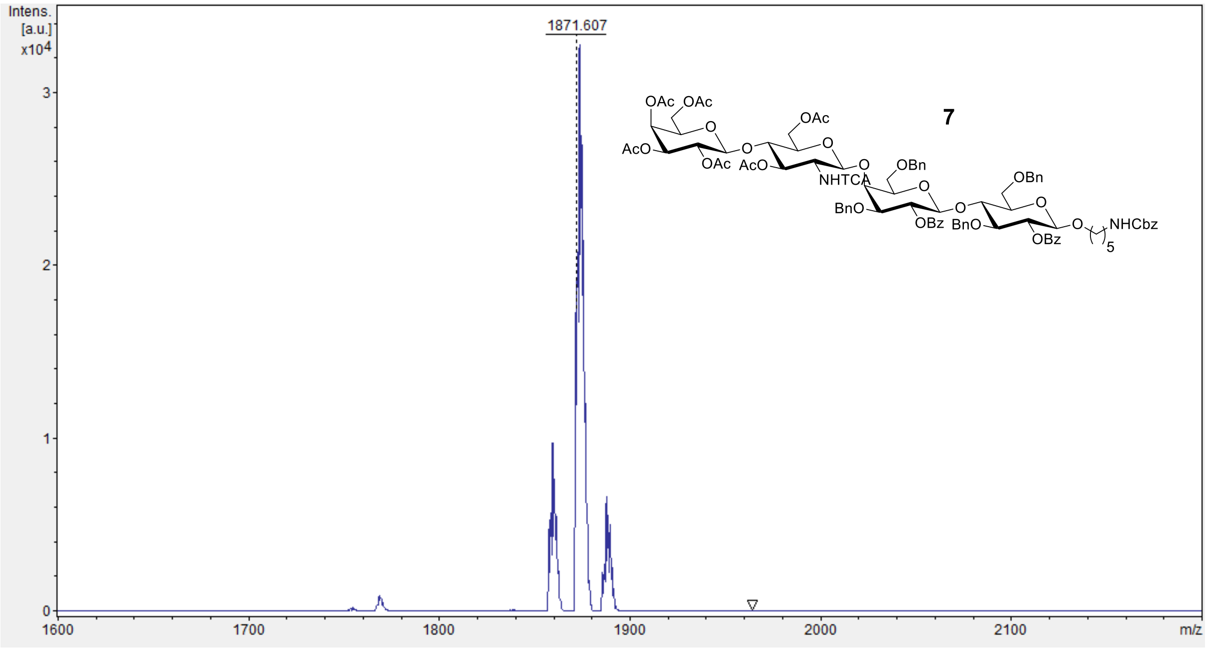
Intens.
[a.u.]
x10^4
1871.607
OAc
OAc
AcO
OAc
AcO
NHTCA
OBn
BnO
OBz
BnO
OBz
NHCbz
5
1600
1700
1800
1900
2000
2100
m/z

7

References

[S1] Eller, S., Collot, M., Yin, J., Hahm, H. S. & Seeberger, P. H. *Angew. Chemie - Int. Ed.*, **2013**, 52, 5858–5861.
